# Staging enmity: reading populist productions of shame with Jelinek’s *On the Royal Road*

**DOI:** 10.12688/openreseurope.15469.2

**Published:** 2023-07-26

**Authors:** Juliane Prade-Weiss

**Affiliations:** 1 Department of Comparative Literature, Ludwig Maximilian University of Munich, Munich, 80799, Germany

**Keywords:** populism, shame, shamelessness, shaming, discourse analysis, Jelinek

## Abstract

**Background**: Populism is often perceived as a shamelessly loud segment of political discourse. However, Jelinek’s play *On the Royal Road*, written on the occasion of Trump’s 2016 election as US president, suggests that populism leads to societal silencing. Jelinek’s text expounds that when a society’s public sphere is marked by ubiquitous enmity against an imagined “we”, grounded in antagonism, then the possibility of speaking to one another disappears, because speaking to one another is based on the willingness to give one’s counterpart space and listen to them. In a public discourse that stages enmity, the counterpart vanishes. Therefore, populism, loud as it is, leads to the silencing of whole communities insofar as they are left with nothing in common but enmity.

**Method**: Critical discourse analysis is used to contextualise close readings of select passages of Jelinek’s play with recent social sciences and humanities research on global populisms to highlight what literary language and the dramatic form can contribute to understanding populism.

**Results**: The silencing populisms entail is fed, in large part, by a dynamics linking the interpersonal emotion of shame to its discursive exploitation in shamelessness and shaming: populist voices transgress rules of democratic debate in the public sphere to elicit outrage by mainstream politics, media, and civil society, which often retort populist shamelessness by shaming populist actors. The audience excitement populist leaders and supporters generate is an important factor in normalizing the emotional, moralizing populist polarization of “us” versus “them” that undermines differentiated discussion and a dispute of arguments.

**Conclusion**: While media and research commonly suggest that with the populist reduction of politics to a spectacle, citizens become a passive audience, the article expounds that audiences play a key role in the production of populist enmity. This insight offers an alley to counteract populism.

## Plain language summary

Populist politicians often appear as a shamelessly loud part of the political debate. Nevertheless, Austrian Nobel-Prize winner Elfriede Jelinek’s play *On the Royal Road*, which she wrote when Donald Trump was elected US president in 2016, suggests that populism leads to societal silencing. Jelinek’s play shows that when the public sphere of a society, notably traditional and social media, are shaped by antagonism and hate between “we”-groups, then the possibility of speaking to one another disappears. Because speaking to one another so as to resolve disputes requires the will to give one’s counterpart space to express their view openly, and listen to them. This becomes impossible when politicians, media, and other public voices shame and shut down their opponent. Therefore, populism leads to the silencing of democratic dialogue and leaves communities polarized. The article combines a reading of Jelinek’s text with recent research on populism from multiple fields to demonstrate that Jelinek’s theatre play can contribute to understanding the phenomenon of populism: it shows that populist speeches and posts on social media function much like theatre plays in that they stage a conflict. A key part of this performance is what I call the populist “shame game”: populist leaders and supporters often consciously transgress commonly accepted norms of speaking in public with the aim of receiving dismissive, shaming responses by mainstream politicians, media, and other public voices. Populists require these dismissive responses to prove their claim that society is split into two groups: unheard voices of the common people they represent, and dismissive elites. The article demonstrates how shaming populist leaders and supporters contributes to polarizing societies and shutting down democratic dialogue, even if audiences who shame populism might do so to defend democracy.

## Introduction

Populism is often perceived as a shamelessly loud segment of political discourse. And yet, Elfriede Jelinek’s 2017 play *On the Royal Road: The Burgher King*, written on the occasion of Donald Trump’s election as US president, ^1^ suggests that populism leads to societal silencing:

We have nothing to say, because no one listens to us anymore, and it’s better that way, we have no more to say, because it’s only us, only us listening to each other, in the book of faces […] ( Jelinek, 2020b, 82)
We fell silent, the more we talk the quieter we are, we find no counter-love, no counterpart, no contrarian, nothing. ( Jelinek, 2020b, 85)
[Wir haben nichts zu sagen, weil uns keiner mehr zuhört, und das ist auch besser so, wir haben nichts mehr zu sagen, weil nur wir, nur wir einander noch zuhören im Buch der Gesichter ( Jelinek, 2020a, 95).
Wir sind verstummt, je mehr wir reden, desto stummer sind wir, wir finden keine Gegenliebe, kein Gegenüber, keinen Gegner, nichts mehr. ( Jelinek, 2020a, 99)]

Not unlike many of Trump’s posts on social media, these passages from Jelinek’s text raise the question: who is the “we” supposedly speaking? Just like with Trump’s posts, the question is not clearly answered in Jelinek. However, their aims differ widely: Trump’s posts offer an imprecise “we” open to identification for many to mobilize a broad audience. *On the Royal Road* advances the suggestion that populists, among whom Trump can doubtlessly be counted ( Jäger, 2022; Wahl-Jorgensen, 2019, 110–128), evoke a “we” that forms a unity in the claim to not being heard by social elites—a “we” based on enmity that only listens to itself inside its social media filter bubble. Therefore, the protagonist of Jelinek’s play— Miss Piggy “as blind seer, with a cane, bleeding eyes, as tradition calls for” ( Jelinek, 2020b, 3), *i.e.*, the tradition of Sophocles’ Tiresias—finds not only “no counter-love”, which is no surprise, but nothing at all, “no counterpart, no contrarian, nothing.” And that is surprising, because populisms of all shades around the globe appear as rhetoric and strategy of introducing opposition and enmity into public discourses. As with many of Jelinek’s more recent texts, *On the Royal Road* features no traditional dialogue between distinct *dramatis personae* but, rather, a continuous discourse. While presented by a single speaker, Miss Piggy, the text is a montage combining heterogenous voices and recurrent speech patterns ( Jelinek & Winter, 1991, 13) so as to dissect the logic of public discourse. With regard to populism—a word never used in *On the Royal Road*, and we will see why—Jelinek’s text expounds a seemingly simple but highly concerning dynamic: when a society’s public sphere is marked by ubiquitous enmity against an imagined “we” that is, in turn, grounded primarily in antagonism towards this adversary, then the possibility of speaking to one another disappears—because speaking to one another is based on the willingness to give one’s counterpart space and listen to them. In a public discourse that stages enmity, the counterpart and contrarian vanish. Therefore, populism, loud as it is, leads to silencing. Not only does it lead to the silencing of populists, but to the silencing of whole communities insofar as they are left with nothing in common but enmity. This silencing enmity is fed, in large part, by a link between the emotion of shame, and its discursive exploitation in shamelessness and shaming.

The question this article addresses is discussed in both social science and humanities research: why and how do current populisms undermine democratic debate and dissent, and lead to a polarization of societies? I take a literary criticism approach, contextualized with social science research, to expound the hypothesis that a cycle of shame, shaming, and shamelessness is a crucial dynamic in this detrimental impact of populism onto societies. While some of the outlined processes pertain to all modes of populism, I shall focus on right-wing populism because it forms the backdrop of Jelinek’s play. I use critical discourse analysis to contextualise close readings of select passages of Jelinek’s play with recent social sciences and humanities research on global populisms to highlight what literary language and the dramatic form can contribute to understanding populism. With this, the article responds to recent research pointing out that “we need to delve deeper into instances of allegedly populist language and discourse in a wide range of contexts” ( Hidalgo-Tenorio *et al.*, 2019, 5). Since prior research has focused primarily on “ideological dimensions” of populisms, the task remains to “consider the more formal or structural dynamics of the debate about populism” ( Goyvaerts & De Cleen, 2020) that contribute to the profound reshaping of societal communication through populisms and allow their ideologies to have a broad impact. The article is based on the hypothesis that reading dramatic texts which negotiate populisms, such as Jelinek’s play, can contribute to understanding the “spectacularisation of politics” ( Hidalgo-Tenorio *et al.*, 2019, 3) in populism because these texts comprise a reflection on the precise linkages between the staging and the contribution of the audience that render a production a spectacle.

In the following, I will expound the communicative effect of populism outlined above—the silencing of societal debated—in a critical discourse analysis approach to Jelinek’s text and multidisciplinary populism research. First, I shall outline why Jelinek’s theatre text can contribute to understanding the socio-political phenomenon of populism, and which methods make this contextualised approach feasible. Second, I will clarify the structure of responsivity in populist discourse. In a third step, I will focus on the interlinkage between shame, shaming, and shamelessness in populism, and, in conclusion, come back to the vanishing counterpart.

## Populism’s theatricality and Jelinek’s discourse analysis

Populism is no coherent theoretical concept or political phenomenon ( Müller, 2016, 1–2). Although populism is “one of the main political buzzwords of the 21st century”, it remains unclear whether it is best described as “as an ideology, a movement”, or “a syndrome” ( Mudde & Kaltwasser, 2017, 1–2). A key reason for populism’s elusiveness is that it is not solely a descriptive concept but “also carries with it an evaluation of the phenomenon it describes” ( van Klink & van der Geest, 2020, 254). In the political arena, populism often serves as a “political *Kampfbegriff* (battle term) to denounce political opponents” ( Mudde & Kaltwasser, 2017, 1). Populist seems to always be the other. While this complicates the term’s descriptive usage, it points to a pivotal dimension of populism that will be in focus of this article: populism is “a media and communication phenomenon” ( Waisbord, 2019) which performs enmity. It is “a communicative strategy that articulates political actors and defines politics as a matter of irreconcilable interests between actors.” (223) In the social sciences and humanities, the communicative aspect of populism has been studied in two major approaches: as discourse and as style ( Nguyen *et al.*, 2022, 150). Both approaches correspond to the form of Jelinek’s text.

In the discourse approach, populism is regarded as part of the “social construction of meaning” in texts and other media that “is not somehow ‘added’ to politics and society” ( *ibid*.) but constitutive of forming both. Methodologically, this approach builds on critical discourse analysis, which is as prominent in empirical social sciences as in literary criticism since it regards “language as social practice” ( Wodak & Meyer, 2016, 5) and views the context of language use as crucial ( De Fina & Geograkopoulou, 2020, 1–7). The aim is to understand the “dialectical relationship between discourse and social reality”: discourse is shaped by surrounding societal realities and contributes to shaping reality ( Hart & Cap, 2014, 1). As an inherently “transdisciplinary, text-analytical approach” ( *ibid*.) to societal reality, critical discourses analysis is the appropriate methodological framework for bringing research on populism into dialogue with Jelinek’s literary text so as to gain structural insights into the communicative dimension of populism. *On the Royal Road* is a literary but (apart from the figure of Miss Piggy) no fictional text; it is, rather, a negotiation of the factual shape of societal communication. Presenting heterogeneous voices that shift between “I” and “we”, as well as speech patterns that dominate political and (social) media discourses, *On the Royal Road* abstains from the traditional dramatic fiction of presenting multiple characters’ actions and emotions on stage ( Vogel, 2013, 48). The play “radically questions the concept of dramatic representation by turning a defamiliarising form of language into the actual protagonist” ( Jürs-Munby, 2020, 46): rendering transparent that the “play” is a reproduction of discourse elements familiar to the audience from media and the public sphere, Jelinek shifts the audience’s attention from the theatrical performance to the public performances she quotes. The blindness of the play’s sole speaker highlights the focus on language: it foregrounds hearing, as Miss Piggy is incapable of looking (back) at the audience that watches her. With the intertextual reference to ancient Greek tragedy in the figure of the blind seer (Tiresias) who speaks the truth (to king Oedipus) as he is not distracted by appearances, *On the Royal Road* also invokes the formal model of the collective voice of the chorus; the historical and formal kernel of tragedy, whose performance is “a ‘speaking out’ more than ‘speaking with each other’” ( Jürs-Munby, 2020, 58). Jelinek’s montage of political, media, and other voices foregrounds key structural dynamics of societal discourse. Therefore, this literary text can contribute to understanding populism as a communication phenomenon when read in context with the discourse approach to populism research. What allows linking Jelinek’s play to studies of different cases of global populisms is that while written on the occasion of Trump’s election as US president *, On the Royal Road* cites the rhetoric of multiple right-wing populisms, notably in Austria and Germany (cf. Jelinek, 2020b 12–13; Jelinek, 2020a, 19–20).

The approach that regards populism as a particular “political *style*” ( Nguyen *et al.*, 2022, 150) highlights how “performative repertoires of populist leaders and their followers interact, and how this affects their relationship” ( Moffitt & Tormey, 2014, 388). This approach focuses on the “inherent theatricality of modern populism” ( Moffitt, 2016, [13]) and thus employs key concepts of dramatic production, such as “performers, audiences, stages and the mise-en-scènes ([157]). Yet, social science approaches to populism rarely clarify the notion of “theatrical production” ( Meyer, 2006, 83) ^2^, or “dramatisation” ( Ekström *et al.*, 2018, 3) achieved by the political style that foregrounds performance, audiences, and stages. As we will see, from a literary criticism perspective, the “theatricality” of populism that social sciences describe can be summarized as staged, *i.e.*, audience-tuned performativity that produces the very enmity it denounces. ^3^ Jelinek’s literary text can contribute to the understanding of populism as style since it negotiates its impact on public discourses not solely in the concepts, but in the actual form of dramatic performance. *On the Royal Road* exhibits the theatrical logic of populist productions by treating populist speech patterns as ready-made elements of a theatre script.

In doing so, Jelinek’s text draws particular attention to the role of emotions in populist discourse. This falls in line with research highlighting that an essential element of populist style is an “increased rhetorical appeal to ‘pathos’ (emotional appeal) as opposed to substantive facts […] to appeal to the people” ( Ekström *et al.*, 2018, 3). Yet, in spite of the research consensus that emotions provide an important breeding ground for populism, “how emotions work in populist framings, narratives, and rhetorics to generate appeal remains unclear” ( Leser & Pates, 2022, 439). In the following, this research lacuna is addressed with regard to the dynamics of shame, shamelessness, and shaming in populism. Approaching populist discourse as production in a comparative reading of Jelinek’s theatre text and research allows us to follow the clue that emotional dynamics in populism are likely “more complex than manipulation or exploitation of people’s sentiments” ( *ibid*.). Analysing the role of emotions in populism, particularly shame, in a communicative approach does not contradict the structure of emotions, *i.e.*, mental states. For while it is impossible to do justice to the multidisciplinary research on emotions in this paper, it does start from the research consensus that emotions are not merely personal, intrapsychic phenomena but fundamentally depend on social contexts, interaction, and negotiation. Of particular relevance to understanding the “theatricality” of populism is that individuals adapt the expression and interpretation of their emotions to social expectations either by “deep-acting”, which seeks to align their mental state with the (implicitly anticipated or explicitly communicated) expectation of others, or by “surface-acting”, which seeks to adapt the expression of personal mental states to these expectations ( Scherke, 2021, 273). Such acting of emotions is not to be misunderstood as phony, it is a crucial interface between individual and communal dimensions of human life. This interface is seminal to the interpersonal emotion of shame, understood as “ *felt* ethic of obligation and regulation that involves an actual or internalized audience that judges one’s thoughts and acts in terms of their relationship to norms and standards that one shares (or is expected to share) with others” ( Locke, 2016, 19). Shame is, in other words, a profoundly theatrical emotion ( Geisenhanslüke, 2019, 15), and it is hence no surprise that it features prominently in populist discourse. Yet, before the interpersonal dynamics of shame can be outlined further, it is necessary to consider the structure of responsivity in populist discourse in general.

## Populist reciprocity

In populist movements, electoral success and political impact often differ: “in many places in the world populists attract a fairly limited number of votes, but, nevertheless, they play a notable role in terms of agenda-setting and policy impact.” ( Mudde & Kaltwasser, 2017, 98) Trump, who won the 2016 elections, is no example to the contrary, as he continues to decisively shape US political discourse in spite of losing the 2020 elections. The “agenda-setting” impact of populisms pertains to both topics and style of the political debate as a whole. Where right-wing populists continuously address immigration in terms of a national crisis, as, for instance, in Germany and Austria, there the purported immigration crisis features prominently even in the election campaigns of mainstream parties. However, populists have a policy impact not only by adding points to the list of pressing issues, their “agenda-setting” reaches further to altering the political style even of mainstream parties ( Ekström *et al.*, 2018, 1) as they add the expression of, and appeal to, emotions to the politically relevant register ( Scherke, 2021, 273–274). Trump thus introduced anger as a factor that is now recognised as “both salient and relevant to political life” ( Wahl-Jorgensen, 2019, 110). Stylistic agenda-setting often includes media, as “[t]he increasing prominence of social media shapes not just the content of mainstream media, but also their affective style” towards a “more extreme, divisive and polarized” presentation (116). Populist agenda-setting, in terms of topics and style of political discourse, thoroughly alters national political cultures, for instance in a “normalization of far-right ideologies, both in content and form” ( Wodak, 2021, 177) that, in turn, impacts international political discourses.

Yet, populist agenda-setting also points out that, as recent research underlines, while populism can have a corrosive impact onto democracies (specifically if it forms alliances with right-wing extremism), it is not, in itself, a pathological aberration from democracy. Populism is, rather, a “permanent shadow of representative politics” ( Müller, 2016, 101) and “the (bad) conscience of liberal democracies” ( Mudde & Kaltwasser, 2017, 116). Populism has a reciprocal relation to democratic mainstream in that it can be understood as a response to its deficiencies, as “illiberal democratic response to undemocratic liberalism” ( *ibid*.). For instance, neoliberal austerity policies of the World Bank, the International Monetary Fund, and the European Union can be viewed as a form of undemocratic liberalism that evoked left-wing populist responses in South America and Europe during the 2010s (102). Yet, populisms cannot only be described as response, they often model themselves as response: as a response to the will of the people, which populisms present as homogeneous and ignored by political elites. This claim is not completely unsubstantial, because mainstream democratic governments are confronted with a conflict “between responsiveness and responsibility”, that is between responding to the will of the populus that they are to represent and the responsibility to observe international treaties ( *ibid*.). This conflict often results in “the general feeling that the political system is unresponsive” (101). Populists offer simple answers to this complex situation, based on an enmity between we-the-people, portrayed as autochthonous, authentic, common-sensical homogeneity and corrupt, perverted elites ( Müller, 2016, 25–31). Conspiracy myths are a structural necessity in the construction of this antagonism because they allow to explain why the supposed will of the majority does not rule in a representative democracy, or (as in the case of Trump’s presidency) why it cannot assert itself unhindered despite an election victory (32).

However, neither populisms’ structural position as illiberal democratic response to undemocratic liberalism, nor populists’ self-fashioning as response to the otherwise ignored will of the people means that populisms offer answers to the political issues they denounce. Populisms might call themselves an ‘alternative’ to the mainstream, such as the prominent German right-wing populist party *Alternative für Deutschland* (“Alternative for Germany” (AfD)), but they hardly propose new policies. The answers right-wing populisms offer—and the paper will henceforth focus on this strain of populisms as it forms the backdrop of Jelinek’s text—paradoxically, often consist in popularising the very principles that underly undemocratic liberalism. On the one hand, *Alternative für Deutschland*, established in 2015, is based on the anti-austerity principle of traditional German post-WWII ordoliberalism, *i.e.*, on the conviction that a “social and economic order based on market principles must be established and maintained by a strong state” ( Binder, 2021, 185). On the other hand, what psychologists Jutta Menschik-Bendele and Klaus Ottomeyer wrote about “recent right-wing extremism and right-wing populism” already in 2002 (305) is no less true for the more recent AfD: they are “based on a brutalisation, ethnisation, and aestheticisation of everyday competition principles”. ^4^ The AfD’s populist response to undemocratic liberalism does, indeed, all but refuse the neoliberal logic of ubiquitous competition in which markets dictate supposed material constrains that often undermine democratic decision-making; the AfD, rather, expands competition into a moralised Social Darwinism ( Butterwegge *et al.*, 2017, 199).

This mimetic relation between undemocratic liberalism and right-wing populist responses is remarkable, given that supporters of populists are often portrayed, in media and research, as losers of modernization who have become disenfranchised in neoliberalism. In view of the outlined mimetic relation, however, they appear as rather emphatic proponents of modern capitalist competition, yet they substitute volatile factors such as economic success by stable criteria like ethnicity and sharply defined gender roles. “Stable social categories may become attractive as a kind of bedrock onto which one can fall back […] This may explain the popularity of right-wing populism both among the ‘losers’ and some ‘winners’ of contemporary neoliberal capitalism” ( Salmela & Scheve, 2017, 585). This, again, points to the difficult fact that while populists and their supporters may form alliances with political extremists, populism is not, in itself, a pathological aberration from social discourse in liberal democracies, but one of its phenomena.

This is true not only in view of (neo-)liberal economic contexts, but for the democratic public in general, even if the public sphere seems to be distorted by populist theatricality. Geffrey Alexander (2021, 3) thus writes: “While these snapshots of democratic drama can be decidedly alarming normatively, empirically they are part and parcel of every civil sphere.” Normatively alarming about populisms of all shades is their “claim to exclusive moral representation” ( Müller, 2016, 23), *i.e.*, the claim to be the only response to, and hence the only legitimate representation of, the will of the people, because this claim leaves no room for a counterpart, or opponent, in public debate. Whoever does not concur with the populist claim of exclusive moral representation is cast not as political opponent, but as an enemy of the people. Populisms hardly ever corroborate empirically that we-the-people they claim to represent is indeed the democratic majority of a populus; the claim is, rather, brought forward with substantial moralisation. Therefore, the populist claim to exclusive moral representation does not merely parasitise the democratic principle of the sovereignty of the people but, indeed, erodes it.

This normatively alarming claim is, still, empirically part of democratic publics because there is a substantial gap between the self-image of the public sphere in liberal democracies and their actual practices. “The empirical operation of actually existing civil spheres is never at one with the normative code of democratic solidarity.” ( Alexander, 2021, 6) Democratic publics are, *de facto*, often scenes of emotionalized controversies, however, they imagine themselves to be fora of peaceful negotiation. And this imagination is necessary, as Alexander (2021, 2) emphasizes because “[p]olitical democracy depends on feelings of mutual regard.” However, as debates on, for instance, civil rights, migration, and gender equality demonstrate, mutual regard of others as political opponents who deserve space and a hearing in the public sphere is never just a given but requires, time and again, shifting the limits of public discourse, *i.e.*, the common understanding of what can, and should, be object of public debate ( Lessenich, 2019). Populisms work on shifting these limits, too, in transgressive statements that foreseeably provoke outcries and excitement because they question the common ground of public discourse ( Schaffrick, 2019, 79–80). However, their aim of shifting the limits of what can be said in public is not to widen the scope of voices and perspectives that are to be heard and considered, but to dismiss those that do not concur. Notably right-wing populists seek to narrow this scope to the point of abolishing the democratic public sphere. This corresponds to the right-wing populist refusal to engage in dialogue, as Ruth Wodak writes: “shamelessness, humiliation of other participants, defamation, lies, and ad hominem attacks dominate.” ( 2021, 177) Dialogue appears unnecessary, of course, and the opponent as enemy, if one claims to represent the morally constituted will of the people against a conspiracy of immoral elites. The difficulty is that the one-sided refusal to engage in civic dialogue has consequences for all sides, populist and non-populist.

These consequences are not comprehensively grasped as the above quoted “agenda-setting and policy impact”. Beyond the normalisation of populist topics and portrayals, populisms’ refusal to engage in democratic dialogue transforms public debates as a whole. The interpersonal emotion of shame and the display of shamelessness are seminal to this transformation because they do not remain one-sided, and render public discourse staged performances of politics. “Elections become more like spectacles than moral performances, empty showcases for staging dramaturgic authority instead of occasions for agonistic display of binding democratic discourse.” ( Alexander, 2021, 4–5) Such criticism of the normalization of populist theatricality is common, but it does not answer the question why, or how, populisms have such a pervasive impact onto the public sphere of societies and its mainstream politics. To approach this issue, Alexander references Sigmund Freud’s insight that the security civilization offers comes at the price of suppressing libidinous and aggressive impulses, wherefore “it is hard […] to be happy in that civilization” (SE21:115). Alexander sees the pleasure in shameless populist theatricality as one of the ways in which the discontent with civilization is uttered, and the suppressed resurfaces—which means that the appeal of populist productions reaches further than populist agendas:

Pornography and violence are standards of popular culture. Extremist populism provides an opportunity for audiences to experience the thrill of evil, to “get beyond” what seems to many the boring and routine banality of the everyday. ( Alexander, 2021, 10)

Populism is exciting, this suggests. Hardly anything provides as rich a material, and as efficient a reassurance of one’s own democratic integrity, as right-wing populism. The same is true from the point of view of supporters of right-wing populism: populist productions are foreseeably met with media outcries and mainstream politics dismissals, and hence a reliable way to confirm the conviction that the political system is elitist and unresponsive. Populist productions evoke a foreseeable mutual indignation that is key to the logic of the populist erosion of the public sphere to the point of silencing. The (often comprehensible) exclusion of right-wing populist positions from public debate reproduces exactly the binarity that populists decry: the antagonism of “we-the-people” against “the elites”. Therefore, some political scientists caution that “[s]trong warnings against extremist forces can backfire” ( Mudde & Kaltwasser, 2017, 112), and so does denouncing populist leaders as pathological ( Müller, 2016, 16–17). This does not mean that mainstream politics, media, and civil society should appease populisms by affirmation. The crux of the matter is, again, a mimetic relation: dismissing populists proves them right since it produces the very exclusion, and the very lacking unresponsiveness, they decry—and since it adopts the populist logic of dominating discourse by shutting down debate. “Calling someone a populist may be a strategic manoeuvre that aims at placing the opponent outside the circle of reasonable people and thus at banning his or her views from the public debate” ( van Klink & van der Geest, 2020, 253). The allegation of being populist can itself be populist ( Dahrendorf, 2019, 6), *i.e.*, a demagogical stopgap for arguments and efficient ways to address deficiencies in democracy.

The mimetic relation of excitement that enfolds between populist productions and audience outcries is the backdrop against which it is relevant that Jelinek’s *On the Royal Road* does not clarify who exactly the speaking “we” is when she writes:

We have nothing to say, because no one listens to us anymore, and it’s better that way, we have no more to say, because it’s only us, only us listening to each other, in the book of faces […]
but I still wanted to report how I was beaten by my father, *okay I beat him too, but we won’t talk about that*, others were also beaten, much worse […], but all that counts for me is what happened to me.
And so, *you see*, because everyone thinks like that, they now have the King they deserve, he beats them every day and they enjoy it, because they deserve it, they deserve entertainment, since they can’t sustain themselves anymore. ( Jelinek, 2020b, 82–83; my italics)
[Wir haben nichts zu sagen, weil uns keiner mehr zuhört, und das ist auch besser so, wir haben nichts mehr zu sagen, weil nur wir, nur wir einander noch zuhören im Buch der Gesichter (…)
aber ich wollte doch noch berichten, wie ich vom Vater geschlagen worden bin, *bitte, ich habe ihn zwar auch geschlagen, aber davon reden wir nicht*, andere wurden ja auch geschlagen, noch viel schlimmer, aber für mich zählt nur, was mir passiert ist.
Und weil alle so denken, *sehen Sie*, weil alle so denken, haben sie jetzt den König, den sie verdienen, der schlägt sie jeden Tag, und sie genießen es, weil sie es verdient haben, sie haben Unterhaltung verdient, wenn sie schon nicht mehr für ihren Unterhalt sorgen können. ( Jelinek, 2020a, 95–96; my italics)]

Jelinek’s theatre text stages the downplaying of the speaker’s own share in the decried conflict, thus undercutting the theatrical stance of self-victimisation (of being unheard, marginalised, and betrayed) that is popular in populisms. However, Jelinek’s text is ambivalent and reaches further than denouncing populist gestures. When Jelinek’s speaker addresses the reader of the play, or the theatre audience respectively, in a parabasis (“you see”), it becomes clear that the unclear “we” does not only include populist voices, following their corrosive claim to speak for we-the-people, but may just as well comprise the audience that reads the play or watches the theatre production of populist theatricality. *On the Royal Road* thus points out that populisms are geared toward the audience of democratic publics that is far larger than the group of possible voters and supporters; it is geared towards an audience whose share in the production of populist theatricality is the pleasure in the provocation of media outrage that so foreseeably answers populist violations of the rules of civic discourse. This does not mean that the corrosion of the civic sphere and democratic structures is a mere show, as Jelinek indicates in the masochistic image of a king assaulting “everyone”, “every day” for their entertainment, thus deviating attention from issues like a lacking income. As Case (2021, 253) notes, “the violent affect in the King’s rhetoric animates the listeners’ loss” and “offers a solution to that loss […] in a satisfying surfeit of violence”. The populist assault on democratic structures and the public sphere is real, yet it is taken as entertaining production in lieu of anything else politics should have to offer. However, this suggests that it is not only populist voices who side-line their share in the exclusion and unresponsiveness they decry, but that media, and media audiences, also tend to disregard the share they have in producing the populist productions they denounce. Jelinek’s text suggests that this disregard is an important factor in the transformation of democracies through populist agenda-setting and policy impact.

This is outlined toward the end of *On the Royal Road*, when the figure of the populist “King” is paralleled with the protagonist of Sophocles’ *Oedipus Rex*, the unknowingly incestuous, patricidal king whose rule scandalises the gods and has, therefore, brought the plague to Thebes. The tragedy is a tension-fraught intertextual reference for Jelinek’s play, since the dramatic conflict Sophocles stages is making the city’s king understand that and why he is the reason for his subjects’ suffering—while, on an intradiegetic level, the blind seer Tiresias knows why, and so does, on a metadiegetic level, the tragedy’s ancient audience that was, most likely, familiar with the myth of Oedipus. Jelinek’s play adopts the principal topic of understanding what goes wrong about political rule, yet the solution it suggests doubts the one Sophocles brings forth: the community’s problems might not only be the “King’s” fault.

So we no longer have anything to say, that’s our punishment and *your joy*. *That makes you happy, doesn’t it!*
[…] The King is to blame for the city’s demise, just because he has come and now he is here. Because he is who he is. The King is guilty, now we know it. *He committed the outrageous transgression of letting himself be voted King by us.* So now he is responsible.
[…] I accuse. I collect my gathered accusations which are falling from the altar I laid myself down on, it’s quite a lot!
[…] but the feelings of hate, resentment, how about those? Directed at him, the King, the one and only, the reconciling sacrifice. ( Jelinek, 2020b, 125–128; my italics)
[Wir haben also jetzt nichts mehr zu sagen, das ist unsere Strafe und *Ihre Freude*. *Da sind Sie aber froh, was?*
(…) Der König ist am Niedergang der Stadt schuldig, allein weil er gekommen und jetzt da ist. Weil er ist, der er ist. *Er hat die ungeheuerliche Übertretung begangen, daß er sich von uns zum König wählen ließ.* Jetzt ist er halt verantwortlich.
(…) Ich klage an. Ich zähle meine gesammelten Anklagen, die vom Opfertisch, auf den ich mich gelegt habe, herabfallen, ist eine ganze Menge!,
(…) die Haßgefühle, der Groll, wie wärs mit denen? Richten sich auf ihn, auf den König, auf den Einzigen, auf das versöhnende Opfer. ( Jelinek, 2020a, 142–145; my italics)]

In these passages, *On the Royal Road* points out the complacency of denouncing populists with populist means. It suggests that indignant laments about populist success, such as Trump’s election as US president, sideline the fact that his election might be symptom of a conflict produced by many, “by us”, *i.e.*, by a (contested) majority of the electorate voting for him as well as by the media outrage and audience attention for his populist productions that have transformed the political field as a whole and have given him a stage. Miss Piggy calls out the audience participation in another parabasis: “That makes you happy, doesn’t it!” The passage’s emphasis on the notion *Opfer* not only, again, cites the role of the victim in which populists like Trump often cast themselves when being accused of corroding democracies. Due to the ambiguity of the German term *Opfer*—which means both “victim” and “sacrifice”—these passages also suggest that with the populist transformation of politics from a discourse into a staging of dispute, representative politics follow the logic of scapegoating ( Lenz, 2022, 198) that lies at the core of ancient Greek tragedy, at least in Freud’s eyes. In *Totem and Tabu* Freud writes:

The Hero of tragedy must suffer; to this day that remains the essence of a tragedy. He had to bear the burden of what was known as ‘tragic guilt’; the basis of that guilt is not always easy to find; for in the light of our everyday life it is often no guilt at all […] The scene upon the stage was derived from the historical scene through a process of *systematic distortion*—one might even say, as the product of a *refined hypocrisy*. In the remote reality it had actually been the members of the Chorus who caused the Hero’s suffering […]. The crime which was thrown on to his shoulders, presumptuousness and rebelliousness against a great authority, was precisely the crime for which the members of the Chorus, the company of brothers, were responsible. Thus the tragic Hero became, though it might be against his will, the redeemer of the Chorus. ( Freud, 1999 SE13:156; my italics)

In the above-quoted passage of Jelinek’s theatre play, such “refined hypocrisy” lies in shifting the responsibility for the populist’s success from “us” to him alone: “He committed the outrageous transgression of letting himself be voted King by us. So now he is responsible.” This shift of responsibility follows the logic outlined by Freud insofar as in *On the Royal Road*, the montage of public voices, speech patterns, and the parabasis mimics the function of the collective voice of the chorus in ancient Greek tragedy. Miss Piggy speaking of “the King” is thus in the position of Sophocles’ chorus, consisting of Theban elders, speaking about the tragic hero Oedipus’ “guilt”. In the Greek text, this guilt is a breach of the genealogic order without any intentional action, or sin in a Christian sense, on the hero’s part. According to Aristotle’s *Poetics*, the hero’s mere “error” ( 1953 1453a 9) gives rise to the audience’s fear, pity, and catharsis that is the psycho-political point of ancient Greek tragic performance (1449b 26–28). Jelinek’s text builds on this classic conception. Yet, it strives not so much for emotional audience participation but, rather, for the audience’s cathartic reflection that their gaze and emotional involvement is the very point of populist productions, and a breeding ground for the success of populisms. It is part of Jelinek’s irony that the “refined hypocrisy” of projecting all the societal polarisation and deficiencies of democracy onto the one populist “King” audiences allowed to rise to power puts this figure into the role of a tragic hero—a role one might hardly associate Trump with, given that his actions are no error but thoroughly intentional. Yet, this irony does not merely serve the common aim of making fun of a populist leader. The irony also offers the insight that what Freud calls “systemic distortion” lies not solely in the populist production but also in the refusal to see the audience’s share in the populist production. It is thus correct, as Case (2021, 251) writes, that “Jelinek’s play mourns the continuation of the King’s reign, asking why it has not been abandoned”, however, it is problematic not to consider the answer the text suggests. Case, rather, concludes her discussion of *On the Royal Road* on a personal note:

I also fail to fully comprehend how Donald Trump continues to claim the presidency of the United States and retain his followers. […] even as the play exhorts the reader/audience to work to understand how the treachery works and to rise up into activist organizing for change, it seems the only explanation for how it continues is in the adoption of operational blindness and narcissism. ( Case, 2021, 255–256)

While the diagnosis of populism’s “operational blindness and narcissism” is certainly not wrong, it stands in lieu of questioning the joy of seeing “the personal and social violence of Trumpism” (249) staged in the production of Jelinek’s play in its affinity to the joy in the outrage about Trump’s populist productions. *On the Royal Road* suggests that such questioning is necessary as the text is sceptical about the gesture of accusing populism: “I accuse. I collect my gathered accusations which are falling from the altar I laid myself down on” ( Jelinek, 2020b, 126). What Rothberg writes about pervasive criticisms of the implication of others in problematic societal structures holds true for the gesture of accusing populists, too: it indulges in “narcissism […] that keeps the privileged subject at the center of analysis” ( Rothberg, 2019, 19). In yet another parabasis, an address to the reader/audience, Miss Piggy spells out the “refined hypocrisy” of shifting all blame onto the figure of the populist that contributes to rendering him the larger-than-life figure (“King”) he claims to be: “He himself, who am I talking about here? If I mean you I should say you, but that makes no sense, you would never listen to me” ( Jelinek, 2020b, 85; cf. Jelinek, 2020a, 98).

Indignant accusations of political opponents as populists partake in staging enmity and thus in the normalization of populist agendas. This is especially true if accusations aim at shaming populist leaders and/or supporters, as I will discuss in the following part.

## Roles in the shame game

Research and media commonly point out fear and hate as the emotional breeding ground for right-wing populism ( Scherke, 2021, 272); linguist Ruth Wodak (2015) even calls it a *Politics of Fear*. From a psychological perspective, “[p]opulism is potent because it thugs at primal fears of being left out.” ( Messina, 2023, 2) The interrelated emotions of fear and hate are evoked in people of quite different status and background who either have already become, or see themselves in danger of becoming, marginalized in neoliberalism, and feel they lack recognition ( Steiner *et al.*, 2022). These people are prone to experience “feelings of fear associated with insecurity, powerlessness, and déclassement on the one hand, and anger, resentment, indignation, and hate, on the other.” ( Nguyen *et al.*, 2022, 147) While status loss is prone to evoke shame in any social and economic order, shame can be regarded “a master emotion in contemporary neoliberal societies” because “within a neoliberal ideology of individual responsibility”, those who experience or fear loss “blame themselves.” ( Salmela, 2019, 181) Shame has recently come into the focus of research due to both the shamelessness populist leaders and supporters often exhibit ( Wodak, 2019; Zembylas, 2021, 19–36), and the insight that shame is what allows fears of marginalization to become translated into hate:

When shame remains repressed and unacknowledged due to chronic impotence, it is transformed into resentment, anger and hatred at alleged “enemies” of the precarious self and associated groups, such as refugees, immigrants, sexual minorities, the long-term unemployed, political and cultural elites, and the “mainstream” media. ( Demertzis, 2021, 43)

Shame is of particular relevance here because it is a relational emotion that feeds well into the theatricality of populism as the experience of seeing oneself lose status in the eyes of others. Shame is painful for individuals as “a primary affect of intersubjective life. We feel shame […] because others matter to us in ways that are constitutive of our social and political identity.” ( Zembylas, 2021, 25). The relationality of shame is a driving force of populism’s corrosive reciprocity outlined above. What I will henceforth call the populist shame game is a conjunction of the emotion of shame individuals feel, calculated displays of shamelessness by populist leaders and supporters, as well as the common practice of mainstream politics, media, and the broader public to shame populist actors. The practice of shaming populist leaders and supporters, for instance as “basket of deplorables” ( Clinton, 2016), is an element of the corrosive dynamics of populism. Because while shame generally functions as a social regulator that incentivises individuals to adhere to group standards, and Sara Ahmed (2014, 101–121) sees shame as a potentially productive emotion that motivates shamed people to take responsibility as community and solicit societal progress, shaming populists does not seem to have a constructive effect. In an interview with the Austrian newspaper *Der Standard*, political scientist Oliver Marchart notes why:

Social shame is an instrument of power. Shaming people is one of the most effective ways to keep them silent because they internalize their subordinate position. […] In recent years, politics have increasingly portrayed poverty or unemployment as self-inflicted. But shaming the so-called right-wing voters as male, white racists has a counterproductive effect. US President Donald Trump has proven that. His offer was: vote for me and you no longer have to be ashamed, because I embody everything you are accused of. I embody utter shamelessness. ^5^ ( Marchart & Hausbichler, 2017, π12)

Cultural theorist Lauren Berlant writes along the same lines:

Trump’s people […] seek freedom from shame. […] People get shamed, or lose their jobs, for example, when they’re just having a little fun making fun. Anti-PC means “I feel unfree.” […] It’s that in capitalism, in liberal society, in many personal relationships, *they* feel used like tools, or ignored, or made to feel small, like gnats. They feel that they don’t matter, and they’re not wrong. ( Berlant, 2016, π10)

This suggests that shaming populist leaders and supporters does not motivate them to align their words and actions to civil sphere standards, but produces displays of shamelessness ( Zembylas, 2021, 20), because shame of (potentially) not keeping up with neoliberal self-made success standards is the reason to support populisms in the first place. In other words, shaming those who feel ashamed of not been able to meet economic group standards offers no way out of the feeling of falling out of line, while populist shamelessness does, even though it changes nothing about the economic reasons for why people (feel they) are unable to keep up. Shameless displays offer a way out of feeling ashamed and blaming oneself because from a psychological point of view, “shamelessness is a reaction formation against shame” ( Wurmser, 1981, 261). More often than not, shame can only be inferred to from the reactions against it because shame gives rise to the wish of not being seen, or, more precisely, of not seeing oneself degraded in the eyes of others. “Shame’s aim is disappearance.” (84) Therefore, shame is, paradoxically, an eminently social emotion while its effect is isolating, and it is a theatrical emotion because it is evoked by a real or imagined audience, although the point of shame is precisely the wish not to be seen. Since shame is induced by an ungracious internalized gaze—by seeing oneself viewed as not keeping up with group standards—shamelessness as re-externalising reaction formation ( Wurmser, 1981, 394) against shame calls for putting oneself self-confidently on display and acting up.

Therefore, the production of shamelessness is a key piece of populist theatricality that provokes outrage and feeds audience excitement. Performances of populist leaders intentionally transgress standards of democratic discourse in the public sphere to elicit the charge of shamelessness from mainstream politics, media, and the public so as to produce the very elitist exclusion they decry. It is doubtful whether the charge of shamelessness can be a productive tactic to counter populism ( Gray, 2018) because it is, indeed, an instrument of power. For unlike the charge of shame *ful*ness, political scientist Jill Locke (2016, 20) notes, the “charge of ‘shamelessness’” marks people or deeds as “outside of the bounds of the agreed-upon social codes rather than a violation of them” and hence “a threat to the social order”. People or actions that are called shameful are, still, regarded as operating within group standards; those that are called shameless are excluded from this group. Locke does not reference populism, ^6^ her agenda is emancipatory, as becomes clear when she further notes:

The Lament That Shame Is Dead […] emerges most pointedly when ordinary people, especially those lacking significant political power and status, resist and refashion the demands of shame and its requirements. It marks as civilization-destroying all political action that self-consciously disavows the terms of shame and reimagines who counts as a citizen and what counts as a civic practice. […] The Lament responds to both moments and movements of increased egalitarianism, periods during which ideals about equality extend into the realm deemed “social” and expose the borders between political and personal, public and private as protective cover for the status quo. ( Locke, 2016, 20)

Many of these points could be true for right-wing populisms, too: they take the marginalization individuals experience or fear from the private to the public realm, they address people who feel they are lacking power and status, and they portray themselves as a democratic upheaval against an undemocratic status quo. However, since the aim of the populist claim to exclusive moral representation is an exclusion of other voices from the public sphere, and hence anti-democratic, it would certainly be naïve to subsume their productions under Locke’s notion of emancipatory shamelessness. Still, accusing populists and their supporters of shamelessness does not guide the way (back) to standards of democratic discourse because it participates in the populist staging of enmity by providing the very elitist exclusion populists denounce.

*On the Royal Road* addresses the relationality of the shame game by commenting on the portrayal of supporters of populism, and the attention paid to their shamelessness:

A horde of young white man say that they are just talking about it, what about?, I don’t understand, whatever, now they are the first ones talking anyway, they always are the first one to take the floor: Listen, say those who would never listen […] they are listened to, *they have our undivided attention, the young white man has taken the stage* […]
The white man answers, but he didn’t understand the question, he does not want any more regulations of speech […] *He is the forgotten working class*, he can see that the elites are also at a loss, this is his hour, now he will control the land […] he will find the ditched ones and hitch them to the masses again […]
Ole King Cole was a merry old soul but hateful and angry too. ( Jelinek, 2020b, 105–107; my italics)
[Eine Rotte junger weißer Männer sagt, daß sie gerade davon redet, wovon?, ich verstehe nicht, egal, jetzt redet sie sowieso zuerst, sie melden sich immer als erste: Hör zu, sagen sie, die nie zuhören würden. (…) man hört ihnen zu, *sie haben unsere ungeteilte Aufmerksamkeit, der junge weiße Mann hat die Szene betreten* (…)
Der weiße Mann antwortet, aber er hat die Frage nicht verstanden, er will keine Sprachregelungen mehr (…) *Er ist die vergessene Arbeiterklasse*, er sieht, daß die Eliten auch nichtmehr weiterwissen, das ist seine Stunde, jetzt wird er das Land beherrschen (…) er wird die Abgehängten finden und sie wieder anhängen an die Masse (…)
Stock und Hut, stehn ihm gut, ja, Haß und Wut auch. ( Jelinek, 2020a, 120–122; my italics)]

In this passage, Jelinek’s text stages the populists’ claim of speaking for the unheard that is gravely at odds with the ample political and media attention given to populist productions. *On the Royal Road* also displays the paradox that while the populist claim to speaking for we-the-people insists on dropping politically correct “regulations of speech” in favour of autochthonous frankness, populists make this claim in highly repetitive speech patterns, demanding hearing, bemoaning a lack of attention, and identifying their supporters as those that were “ditched” by the establishment. However, this passage from Jelinek’s text, yet again, also points out the part of the audience in the populist production: “they have our undivided attention, the young white man has taken the stage”. The latter might seem like a stage direction, however, “the young white man” is not an individual character of Jelinek’s text, which features no distinct *dramatis personae*, but the stereotypical supporter of right-wing populism whom Marchart and Berlant reference, too. Since *On the Royal Road* is a continuous discourse rather than distinct voices of *dramatis personae*, it is impossible to decide with certainty whether “[t]he white man[’s]” perspective is voiced in the claim “He is the forgotten working class”. It might just as well be the media and research view of the paradigmatical supporter of right-wing populism, given that the opening of the passage (“they have our undivided attention”) appears to reflect an audience perspective. And this indecisiveness is the poetical point of Jelinek’s negotiation of populist discourse, which consists of both the populist staging of being excluded from mainstream politics and the audience’s attention to that claim. Populist leaders and supporters assume a shameless posture, and reproduce transgressive speech patterns, in order to command the audiences’ attention. Both the populist actors’ and the audiences’ perspectives together constitute what is called a role, in theatre as in social life. Social roles are formed in the interplay between group expectations and individual expressions that answer to them—even transgressive populist productions answer to group standards insofar as they are calculated to cross expectations about how to act in political discourse and the public sphere. “[T]he young white man” who supports right-wing populism is a social role, produced by both the transgressive populist staging of politics and the outraged, often shaming portrayal of populists in media and mainstream politics. *On the Royal Road* emphasises this character of a role by introducing the emotions of hatred and anger, which are commonly associated with populism, in the words of a popular song. This suggests that “the young white man[‘s]” lines are standardised, which grants him attention.

This poetical approach to populist theatricality adds an insight to its discussion in research: Jelinek’s play highlights that the self-portrayal and the media portrayal of right-wing populism supporters are intertwined and follow the logic of a theatrical role. This points out that although shameless populist productions intent to break the rules of civic discourse in democracies, and although the answering “charge of ‘shamelessness’” marks people or deeds as “outside of the bounds of the agreed-upon social codes” ( Locke, 2016, 20), populist productions have become well-embedded in public discourse. *On the Royal Road* suggests that whoever assumes the role of “the young white man” displays deep- and/or surface-acting to align himself with public expectations of the populist breach of democratic rules; he answers audience expectations of what transgressions look and sound like. This does not mean that Jelinek’s text portrays the hatred and anger populists produce as “forced upon” people “from the outside” ^7^ ( Felber, 2019, 344). *On the Royal Road*, rather, shows that the populist spectacle of shameless transgressions is not at all beyond the limits of the public sphere but a routine well-embedded into audience expectations. Jelinek’s text thus suggests that it is not merely a hostile few who threaten democracy as populists, but that it is undermined by a routine performance before everyone’s eyes and due to the excitement of large audiences that gives populists a stage.

Jelinek’s emphasis on theatrical social roles in populisms pays heed to the fact that although it is doubtlessly necessary to understand the fear and shame underlying populist anger and hate, the paradigmatic figure of “the young white man” is not a good object for a traditional hermeneutics of *Einfühlung* (empathy). First, because the role aims for surface effect, seeks to challenge audiences, and averts empathy by acting hostile and violent: “he says a lot more people will simply disappear through violence. Whatever he can contribute to it, he will do.” ( Jelinek, 2020b, 105; cf. Jelinek, 2020a, 121) Second, because displaying comprehension for “the young white man[‘s]” emotions easily feeds into the populist production of enmity as elitist condescension. It can, indeed, be condescending to call supporters of right-wing populism “left behind” ( Zembylas, 2021, 20), or “losers of modernisation” ( Lengfeld, 2017), because such diagnoses offer the tempting opportunity for observers to situate themselves as keeping up with the crowd, and on the winning side of modernisation—while the global climate crisis could raise the question whether there are any true winners of the economic modernisation of the planet. Condescension is also easy to see in a further standard explanation for why people support right-wing populisms:

[…] reactionary populisms […] appeal to certain kinds of people; reactionary ideologies and movements resonate with people who have underlying authoritarian character structures that willingly reject factual evidence and/or logic. ( Langman & Schatz, 2021, 168)

From a social psychology point of view, the theory of an “authoritarian character” explains little about broad populism support but, rather, fulfils an “order function” for the theorist, whom it grants a “defence against fear and confusion” ^8^ ( Menschik-Bendele & Ottomeyer, 2002, 18). Jelinek’s montage of recurrent speech patterns that shape the discourse both of and on right-wing populisms draws attention to this entanglement of different positions and to the relationality of emotions populisms evoke. *On the Royal Road* stages the populist hostility against dialogue as well as the dismissal of populist actors by mainstream politics, media, and the broader public. Both hinge on shame: on shame people seek to overcome by shameless transgressions which, however, reinforce the shame as the public retorts transgressions by shaming—which must then be overcome by more shameless displays. A key effect of this shame game is that the hateful young white man, who features so prominently in the criticism of populism as ashamed about his low social status, appears less than a comprehensible counterpart or political contrarian but, rather, as an exciting figure of transgression, hatred, and anger, that is to say: as a theatrical role.

This dynamics of shame points to an intricate reciprocity: hatred and enmity are seminal elements of populisms, especially right-wing populisms, since the claim to speaking for we-the-people requires the exclusion, and denunciation, of others who do not belong to this homogenous “we”. However, the term populism is used not only for descriptive purposes, but also as a denunciation that stirs up enmity towards opponents. The difficulty is that the transgressive, hostile rhetoric of right-wing populists as well as the indignant shaming of their supporters in response stages political debate but undermines the dialogical structure of democratic discourse since both do not aim for exchange. Jelinek’s *On the Royal Road* shows this logic of populism but eschews the battle term *populism* so as not to, one may conjecture, participate in the polarization it entails. Avoiding participation in populist polarization is not easy because populist productions seek to rule out the possibility of distanced observation and differentiation. This is why shamelessness is a key part of populist productions: the shame game—the performance of transgressions that foreseeably provokes outrage and accusations of shamelessness—seeks to involve everyone on an emotional level in the production of a binary, we-the-people versus the elites view of society. Populist shamelessness seeks to force everyone to position themselves on one of the sides, to be either scandalized by or supportive of the transgression. In short: populisms aim to leave no room for not being excited.

Transgressive populist rhetoric is corrosive to democratic dialogue in the public sphere because it seeks to dictate possible audience responses so as to rule out any position that does not confirm the claim that society is split into the binarity of we-the-people versus dismissive elites. Populist productions that harp on shame thus not only assume as transgressive role to secure attention, but they also allocate restrictive roles to all other social actors. I want to outline this in a brief look at a populist production of shamelessness before outlining how the shame game makes the position of the counterpart vanish and leads to societal silence.

The dynamics of the populist shame game becomes particularly clear in debates about cultural memory (politics) because “cultural trauma” is “a social force that primes citizen-audiences to welcome extreme populism” ( Alexander, 2021, 10). Populists often address elements of a pain- and shame-ful past, such as National Socialism in Germany, even if memory politics is not the subject matter of the debate. In a contribution to the 20 November 2020 parliamentary debate in the German *Bundestag*, Member of the German Parliament Karsten Hilse, ^9^ who is also member of the right-wing populist party AfD, evokes National Socialism in a debate about something quite different: the issue at hand is that the previous day, guests the AfD had invited to the parliamentary vote on an anti-Covid-19 measures law harassed and threatened representatives of other parties.

The opening of Hilse’s speech is in line with the social and parliamentary expectations about how to respond to these transgressions: “I condemn in the strongest possible terms that guests abuse their hospitality rights” ^10^ ( *Dass Gäste ihr Gastrecht […] missbrauchen, verurteile ich auf das Schärfste*; Hilse, 2020, 0:17–0:23). After this initial gesture, he immediately shifts his focus away from the transgression of guests of his own party toward accusing all other members of the parliament of a far graver transgression: “This Wednesday was—but not because of this, because of the abuse of hospitality rights—a historic day” ( *Dieser Mittwoch war—aber nicht deswegen, wegen der Missachtung des Gastrechtes—ein geschichtsträchtiger Tag*; 0:31–0:37). The day was historic, Hilse continues, because the German parliament has voted for a law that is “by some also” called “Enabling Act” ( *von einigen auch Ermächtigungsgesetz*; 0:47–49). Mentioning this term stirs up unrest in the audience in the room because in German memory culture, it is reminiscent of the Enabling Act of 1933 by which the parliament gave the National Socialist government the authority to make and enforce laws without any involvement of the parliament, thus legitimising authoritarian rule. Calling a 2020 law about anti-Covid-19 measures an “Enabling Act” is a transgression not only because it denounces the modern act, but even more so because it extenuates the gravity of the 1933 act. Hilse is aware of this transgression, hence he concedes “and yes, the word Enabling Act has an explosive connotation” ( *und ja, das Wort Ermächtigungsgesetz hat eine brisante Konnotation*; 0:49–0:55). However, he goes on to explain that it had been a standard term ever since 1914, and only received its negative connotation after the 1933 act which “unhinged democracy and resulted in the most severe catastrophe of the twentieth century” ( *die Demokratie aus den Angeln hob und zur schlimmsten Katastrophe des zwanzigsten Jahrhunderts führte*; 1:27–1:32).

Hilse’s manoeuvre of shaming the parliamentary majority can be called shameless because it fashions itself as critical reconstruction of a conceptual history but omits the fact that contemporary German politics act in the aftermath of National Socialism and cannot return to before 1933. In the German public, referencing National Socialism is a reliable way to emotionalise debates and to elicit outrage—outrage that, in this case, deviates attention from the actions of the AfD members and their guests the previous day, which illustrate that the right-wing populist party is very likely and consciously posing a threat to representative democracy. Hilse, rather, commands the parliament’s attention to focus on a shame in which, according to the post-reunification memory culture consensus, all Germans share. He invokes the *topos* of German political rhetoric that National Socialism is the *ex negativo* basis of political responsibility in the present. Hilse appropriates the mainstream “politics of shame (at the German past) and pity” (for the victims of National Socialism) that many political and civil society actors use “to differentiate themselves from the populist and the extreme right” ( Eckersley, 2020, 229), for the very populist shame game. While he pays lip service to the German memory culture consensus in mentioning “the most severe catastrophe of the twentieth century” without explicitly naming the Shoah, he breaks one of the fundamental rules of this consensus by analogizing a current phenomenon with National Socialist violence in calling the law about anti-Covid-19 measures an “Enabling Act”. Hilse does so in order to claim the position of historically informed political responsibility exclusively for the AfD, thus laying the ground for the key populist claim to exclusive moral representation of the people.

The previous day, Hilse continues, was historic because never before in post-war Germany had there been so much “resistance” ( *Widerstand*) against a parliamentary act ( Hilse, 2020, 2:18–2:39). This is a continuation of the leitmotif of historical responsibility because “resistance” invokes the positively connotated but factually sparse historical resistance of Germans against National Socialism. He claims the position of responsibility and resistance against a supposedly authoritarian majority on the grounds that “we”, the AfD members of parliament, had been the only ones to respond to the many protest messages from the people, and thus “clearly positioned ourselves for liberty and democracy” ( *uns klar positioniert für Freiheit und Demokratie*; 2:40–2:24). Hilse’s strategy is thus to reverse the media and research discourse on the right-wing populist AfD, and to accuse all other parliamentary parties of being a threat to democratic freedom. This implies the claim that all others have been unresponsive to the supposedly homogenous voice of the people that spoke out against the passed anti-Covid-19 measures. Articulating the fundamental populist claim to respond to the otherwise unheard voice of we-the-people, and thus being their only legitimate parliamentary representation, Hilde’s shaming manoeuvre culminates: 413 members of the parliament “have [...], out of opportunism or out of cowardice, voted in favor of a law that throws open the door to democracy. Shame on you!” ( *haben […] aus Opportunität oder aus Feigheit für ein Gesetz gestimmt, das die Tür zur Demokratie aufstößt. Schämen Sie sich!* 2:53–3:03) Appealing to shame is a narrative means for “the construction of antagonistic characters” ( Langa & Ilundian, 2019, 30). Yet even in German, Hilse’s image of “throwing open the door to democracy” is oddly ambivalent as it usually connotes a progressive movement (such as involving more people in political processes), not a threat, as Hilse insinuates. It is impossible to tell whether the image is a mishap or intentional populist vagueness to render the threat more threatening. What is unambiguous is Hilse’s strategy of responding to the transgression of inviting guests who harass and threaten members of parliament by counter-shaming the plenum. Members of the parliamentary majority, he suggests, should be ashamed of themselves because by voting for the anti-Covid-19 measures act, they have reproduced the 1933 authorization of a criminal state to rule over the supposedly resistant German people. While these historical references might appear far-fetched to a distanced observer, Hilse’s speech answers to the public’s expectation of what right-wing populist transgressions look like (they trivialise National Socialism) so as to allocate a precisely defined role to the parliamentary and media audience: they contribute the excitement without which populisms is naught. Hilse’s speech successfully produces the other party’s dismissive stance which his speech decries when his “Shame on you!” (3:03) is immediately answered by an identical call from the audience: “Shame on you!” (3:04). Remarkably, Hilse also manages to direct the camera work of the neutral parliamentary TV: as soon as his “Shame on you!” is echoed, the camera switches to a view of the audience (3:04) that plays an essential role in the populist staging of enmity in the shame game.

Admonishing others that they should be ashamed of themselves while committing a shamelessness oneself is not only an interesting rhetorical turn but grants insight into both the reciprocity at the core of the shame game elaborated above, and the destruction of the position of the counterpart in populism.

## The vanishing counterpart

The problem about the populist shame game—that is, transgressive populist productions that foreseeably elicit accusations of shamelessness—is that it renders the public a *mere* audience and makes the stance of a counterpart disappear that is indispensable for democratic dispute. This issue reaches further than the observation that when “politics is turned into a spectacle”, it “reduces citizens to the passive role of spectators” ( van Klink & van der Geest, 2020, 256). In populist productions, the audience is everything but passive since its outrage, excitement, dismissal, and counter-shaming are necessary to display the binarity of we-the-people versus condescending elites that populists decry. The issue is that the emotional dynamics of the shame-game are very often successful in ruling out any other response. I will outline this by drawing on (social) psychology. The point of this approach is not to pathologize populist leaders or supporters but to get a grasp of the communicative effects of shame, shamelessness, and shaming that could explain in detail why, and how, shame that “remains repressed and unacknowledged due to chronic impotence, […] is transformed into resentment, anger and hatred at alleged ‘enemies’ of the precarious self and associated groups” ( Demertzis, 2021, 43).

Shamelessness, I had outlined above, “is a reaction formation against shame” ( Wurmser, 1981, 261). Shamelessness wards off shame in a “defiant display of ‘power’” that is “undaunted by fear of ridicule and mockery” ( Wurmser, 1981, 260). This power is at stake in Hilse’s transgression of German memory culture standards in a parliamentary speech: his maneuver of counter-shaming the parliamentary majority aims to ward off a potential degradation in their eyes (over having permitted transgressive guests into the parliament) by degrading the audience, by disdaining their norms and consensus. Therefore, his display of shamelessness stirs up outrage in the plenum. His call “Shame on you!” ( Hilse, 2020, 3:03) reverts the sites of the conflict at hand by claiming that the AfD, not the parliamentary majority, is, in fact, the normative authority in the eyes of which all other parliamentary parties have degraded themselves. Of course, I cannot know whether Hilse is actually ashamed that guests of the AfD have harassed and threatened other parliamentary representatives; I would, in fact, deem it rather unlikely. Or whether his transgressive speech seeks to ward off the impotence of being immutably bound to the atrocious Germany history of National Socialism and having to answer for it. However, populist actors do not need to actually feel shame in order to assume the role of the angry white man and elicit the corrosive communicative effects of the shame game that permits no response apart from being exciting by the production, no response that cannot be fed into the polarizing logic of we-the-people versus dismissive elites. The shame-game reliably elicits a confirmation of the decried social binarity because responses to populist shamelessness are reaction formations against shame, too. The call that immediately echoes Hilse’s “Shame on you!” (2020, 3:04) can be understood as an attempt to ward off the shame the caller in the plenum feels, but it succumbs to the logic of counter-shaming Hilse’s speech dictates.

Psychologist León Wurmser (1981, 208) lists three reaction formations against shame in general that are less obvious than counter-shaming: “depersonalization, mysteriousness, and shamelessness”. Besides, and often in connection with, shamelessness, mystification is a further strategy parliamentary members of the AfD employ in transgressions of the German memory culture consensus, for instance Alexander Gauland, who stated in a 2018 party meeting: “Hitler and the Nazis are just a piece of birdshit in more than 1000 years of successful German history.” ^11^ ( DW, 2018, π1) This trivialization of National Socialism is, at the same time, a piece of mystification as it links the Nazi propaganda myth of having ushered in a Thousand-Year Reich to the neoliberal paradigm of success. Depersonalisation, on the other hand, is a reaction formation against shame that can be seen in the audience of Hilse’s parliamentary speech. The parliamentary camera catches the reaction of representatives of other parties to his call that they should be ashamed of themselves ( Hilse, 2020, 3:06): of the three members of parliament whose faces are captured on camera, none looks at Hilse; two look away, one hides her eyes behind her hand, as if not to be present, not to witness the transgressive speech they are ashamed of. Depersonalization in reaction to feeling ashamed by populist shamelessness bars audiences from responding to transgressive populist productions in a differentiated manner that does not fall in line with the dynamics of the shame-game. An audience that hides in depersonalization cannot be a counterpart or contrarian in a democratic debate but confirms the populist claim that the political system is unresponsive.

The depersonalizing effect of the shame game that makes the position of the counterpart vanish corresponds to two further defense mechanisms against shame that have recently been highlighted in populism research: first “ *splitting*”, the operation of “compartmentalizing good and bad qualities in themselves and others”, the psychological foundation of the binarity of we-the-people against the elites; and second “ *projective identification*”, the operation of disposing of “unwanted aspects of [oneself] by projecting these intolerable qualities or characteristics onto another person or other people” ( Messina, 2023, 2–3). Projective identification is what translates shame into hate by outsourcing the shame ( Watkins, 2018, 31) and shifting everything that causes shame, such as a low social status, onto others, such as marginalized groups or elites that supposedly give political preference to those groups. Reaction formations against shame can be fostered in the populist staging of enmity because they are unconscious. Conscious and intentional populist “blame-shifting” ( Messina, 2023, 3) appears compelling to many because it builds not primarily on extremist convictions, but on the conventional impulse to escape shame by freeing oneself from shame-inducing qualities and shifting them onto someone else. Reaction formations against shame are complex because they happen as much in the psyche of individuals as between actors ( Scherke, 2021, 275). Yet, since the projection of individuals or groups as located outside, and hostile towards, we-the-people is a function of safeguarding a self-image free from shame, these others are no counterpart, or contrarian who could respond, but an image, a stereotype. While projections that seek to ward off shame name actually existent social groups—“such as refugees, immigrants, sexual minorities, the long-term unemployed, political and cultural elites, and the ‘mainstream’ media” ( Demertzis, 2021, 43)—identifying them does not serve the aim of comprehending them, but of tying unwanted qualities of the self to someone else. The same is true about the role of “[t]he young white man” ( Jelinek, 2020b, 105) who is ashamed of being marginalized in neoliberalism: although there are, of course, many angry young white man wo shamelessly stage their support of right-wing populists, the role that the figure of “the young white man” plays in many criticisms of populisms is not that of a possible interlocutor, but a projection that fulfils a psychic function for the critic. This figure has “our undivided attention” (105) and, still, never listens because his role is something else: he carries the hatred and the anger that cannot be acknowledged by someone who wants to be outraged about right-wing populism with the good conscience of taking the stance of democracy. What a community shaped by populism has in common is the atomized, non-dialogical staging of enmity.

The mobilization of reaction formations against shame in transgressive populist productions of shamelessness is corrosive to democratic dialogue because it reduces those who are outside we-the-people to mere images, and elicits responses that are structured similarly. The populist shame game avoids being confronted with the fact that the images is creates do not correspond to the complexity of individuals or social groups because it provides only for restrictive possibilities to respond, producing an audience who confirms the binary model of society no matter whether it agrees or reacts with shaming.

The dynamics of depersonalization, splitting, and projective identification, which all seek to ward off shame, make the position of the counterpart and contrarian disappear in a public discourse that stages enmity in the shame-game. Jelinek’s *On the Royal Road* points out this disappearance, and the consequent breakdown of communication:

Oh, that you humans feel no shame! But why should we be ashamed, he did not set fires or paid for murders, thus we chose him in full possession of [our] powers, but he not us. […]
*You should be ashamed! I have no idea whom I mean.* Why should we be ashamed, say the kids. They don’t see the trouble in store for their clan. ( Jelinek, 2020b, 43; my italics)
[Daß ihr Menschenkinder euch nicht schämt! Aber warum sollen wir un schämen, er hat ja keinen Brand gestiftet und keinen Mord bezahlt, und so haben wir ihn im Vollbesitz unserer Kräfte erwählt, er uns aber nicht. (…)
*Schämen Sie sich! Ich habe keine Ahnung, wen ich meine.* Warum sollen wir uns schämen, sagen die Kinder. Das Schlimme, das ihren Stamm betreffen wird, sehn sie nicht. ( Jelinek, 2020a, 53; my italics)]

In this passage, Miss Piggy voices the Cassandra-like futility of the seer who spells out the wrongful deed but, still, lacks the means to move anyone to change course. She lacks these means because, as voice that presents populist discourse, she cannot assume a position beyond the roles it prescribes. When she voices an attempt at shaming supporters of populists (“Oh, that you humans feel no shame!”), she does not elicit insight into the destructiveness of supporting populisms because her shaming follows the same logic as populist discourses. Jelinek’s text spells out that the dynamics of the shame game makes the position of the counterpart disappear when the voice calls out—just like Hilse and the echo from his audience—“You should be ashamed!”, and comments, “I have no idea whom I mean.” This suggests that while it is not hard to see that the corrosion of democratic debate in populism is a shame, this very corrosion makes the position of the interlocutor disappear who could be addressed so as to do something about it together. Therefore, Miss Piggy later states, “we find no counter-love, no counterpart, no contrarian, nothing.” ( Jelinek, 2020b, 85) The loud dispute staged in both populist shamelessness and audiences’ counter-shaming is, paradoxically, tantamount to the silencing of whole communities insofar as they are left with nothing in common but enmity. This is the structural reason for why *On the Royal Road* features no conventional distinction of roles and voices: while populisms rely on a mimetic reciprocity between “us” and “the others”, the shame-game rules out communicative relationality, *i.e.*, a dialogue or even dispute with, and comprehension of, someone who is more than an audience, more than an image supporting one’s self-image, *i.e.*, someone else.

What is to be done about the vanishing of the counterpart in populism? While research proposed contradicting the logical and ethical implications of populist claims in a number of different approaches, such as addressing the societal problems they name but rejecting their framing ( van Klink & van der Geest, 2020, 258–268), the verdict about the shame game is clear: “This trading in shame by the Right and the Left is dangerous and needs to be refused.” ( Watkins, 2018, 34) This is easier said than done as reaction formations against shame, such as depersonalisation in the audience evoked by populist transgressions of fundamental rules of democratic debate, are unconscious. Interrupting unconscious dynamics requires reflection. From a psychological point of view, interrupting the interplay between shame, shamelessness, and shaming so as to counter the production of a binary society, split in “us” and “them”, requires acknowledging one’s own ambivalent emotions so as to work through shame ( Watkins, 2018, 33). Social psychology approaches to historical trauma that “primes citizen-audiences to welcome extreme populism” ( Alexander, 2021, 10) suggest the same working through shame, *i.e.*, acknowledging what induces shame and resisting the defence mechanisms of splitting and projective identification which clear the self-image of shame-inducing qualities and shift them onto someone else. The aim of this reflection is to recognize that destructiveness in to be found not only in others, but also in one’s own actions ( Gobodo-Madikizela, 2021, 31). In the context of the shame game, this could lead to recognizing that introducing destructive shaming practices into the public sphere is not confined to populists who portray society as hostile binarity of we-the-people *versus* the elites. And to acknowledging that outraged shaming responses, even if given with the best of democratic intentions, contribute to corroding democratic dialogue by confirming, and thus producing, the populist binarity in painting stereotypical images such as “the young white man” full of anger, hate, and shamelessness.

Such differentiated reflection that acknowledges the audiences’ contribution to populist productions is not the same as Trump diluting right-wing extremist violence against counter-protesters at a white supremacist rally in Charlottesville by stating: “I think there’s blame on both sides” ( Trump & Drobnic Holan, 2019, π55). It is not the same because his purported differentiation is a blame-shifting that follows the logic of projective identification to find culprits somewhere else than among his supporters. Working through shame means more than finding “blame on both sides”, and more that identifying oneself as contributing to one of these sides. It further means realizing that the assumption of only two sides is wrong as it cannot capture the complexity of voices and positions of civic society; that the binarity of a rally versus counter-protesters emphasised by Trump, or the right-wing populist party versus all other parliamentary parties staged by Hilse, are forms of democratic dispute, but not its entirety.

*On the Royal Road* proposes reflecting on the complexity of civic discourse in staging one continuous discourse rather than populist versus anti-populist characters, and in shifting between the pronouns “I”, “we”, and “they”, so as to undermine the dramaturgical binarity of stage *versus* audience. The third person plural can often be read as referring to very different political actors, and to both sides of the populist binarity. The play thus suggests that the staging of enmity in populism is based on the dynamics of splitting and projective identification not only in populists, but also their civil society audiences. *On the Royal Road* draws attention to this corrosive interaction in the civil sphere in a reflection on the role Jelinek’s writing and her audience play in populist productions:

[…] it’s *her* favorite activity, rail at people, blame them, accuse them, stupidly imitate the spazzes, who else will do it? […]
The no-show of the hoped-for satisfaction that anyone would still listen to *me*, to us Dichter und Denker, the poets and thinkers […] does surprise me. In fact, it was firmly promised to us. But once *again only a few were left in the house to watch with schadenfreude*, how this satisfaction isn’t coming, it always is interrupted like my wireless ( Jelinek, 2020b 78–79; my italics)
[(…) das ist ja *ihre* Lieblingsbeschäftigung, Menschen beschimpfen, anklagen, beschuldigen, blöd nachahmen die Spastis, wer soll das denn sonst machen? (…)
Das Ausbleiben der erhofften Befriedigung, daß *mir*, daß uns Dichtern und Sagern noch wer zuhört, das wundert mich jetzt schon. Eigentlich wurde es uns fix versprochen. Es sind aber *wieder nur wenige im Saal übriggeblieben, um schadenfroh zuzusehen*, wie diese Befriedigung schon wieder ausbleibt, die bricht immer wieder ab, wie mein WLAN ( Jelinek, 2020a, 92–93; my italics)]

It is unclear to whom the pronoun “her” refers in this passage; it could be “Mrs. King”, the populist’s presidential wife, that is mentioned before ( Jelinek, 2020b, 78; cf. Jelinek, 2020a, 92), Miss Piggy herself, or Jelinek, with whom the charge of “stupidly imitat[ing]” would stick in terms of poetical and theatrical representation. That the latter two might be comprised in “her” is suggested by the following elaboration (that shifts to the first person singular) on the disappointment about the lacking public impact of literature and culture. This disappointment might, of course, be read as irony, however, such a distancing reading would undercut the remarkable portray of the audience’s role in the representation of populist discourse: “again only a few were left in the house to watch with schadenfreude”, taking pleasure in the adversity someone else faces. This can be read as a comment on Jelinek’s role as a public figure and political voice whose texts often criticise recent political events and who, therefore, often faces a hostile audience. In 1995, the Austrian right-wing populist party Freiheitliche Partei Österreichs (FPÖ) asked on one of its election posters: “Do you love Scholten, Jelinek, Häupl, Peymann, Pasterk … or arts and culture?” ^12^ ( Der Standard, 2004) At the same time, bringing up the audience’s “schadenfreude” questions the stance even of a friendly audience that shares in Jelinek’s concern about populism: why watch the production of belligerent populist discourse—if not for the satisfying excitement it provides, the “schadenfreude” in seeing Jelinek take on Trump and other populists, “blame them, accuse them, stupidly imitate” them. Yet, regarding populism as source of excitement, pleasure, and satisfaction—not least with one’s own democratic stance—that never runs dry is a condescension which feeds well into transgressive populist productions and normalizes staging enmity. The turn of Jelinek’s play against itself is a poetical reflexion in which *On the Royal Road* acknowledges the destructiveness that lies in its own representation and dramatic production. In interrupting the portrayal of populist discourse to point out that the play does not seek to provide a cheap laughingstock and the easy confirmation of a good conscience, Jelinek’s play also solicits the audience to reflect on its contribution to populist polarisation and breakdown of communication.

*On the Royal Road* thus provides the insight that staging enmity corrupts dialogue—first, because it fosters the production of a clear-cut conflict rather than a differentiated portrayal of complexity, second because it suggests that the audience is a mere passive consumer rather than an active participant in the production. Jelinek’s play can be understood as seeking to produce what social psychology research suggests: “disidentification” ( Horn, 2021, 182) with ready-made collectives so as to acknowledge one’s own destructiveness, and to approach others as counterparts, or contrarians, in a dispute rather than stereotypes that confirm one’s convictions.

Literary texts are no polls that show the broader impact of populist discourse onto societies. However, as I have tried to outline, a contextualised reading of Jelinek’s play can contribute to the multidisciplinary understanding of the communicative effects of populism by expounding the seminal role of media and public audiences in the populist rendering of politics as spectacle that undermines democratic debate. The cycle of shaming populists that is foreseeably answered by populist shamelessness reduces dissent to enmity. Outlining the dynamics in which emotions of the wider public feed populist productions is an important yet, as Landweer puts it, under-addressed aspect in understanding populism and other phenomena that are currently corroding liberal democracies: “contempt” at populists, she writes, is generally not seen as problematic, “but is instead normatively promoted as an apt feeling associated with liberal orientations. There is no public awareness of its hidden aggressive tendencies. A first step toward acknowledging this danger might be, somewhat ironically, to discover and acknowledge one’s own contempt for fanaticism, populism and related phenomena.” ( Landweer, 2022, 154). Jelinek’s *On the Royal Road* promotes such reflection as it confronts audiences with their role in populism. The play suggests that shaming populists is everything but the royal road to limiting the detrimental effects of populist discourse—audiences’ joy in the spectacle of shame-game, on the contrary, feed into the populist production of enmity.

## Ethics and consent

Ethical approval and consent were not required

## Data Availability

No data are associated with this article

## Footnotes

^1^ Felber, 2019, 344; the English translation premiered 27 March 2017, at the NYC Segal Theatre Center.

^2^My translation of: *theatralische Inszenierung*.

^3^Cf. the concept of performativity in Butler (1993, 2): “performativity must be understood […] as the reiterative and citational practice by which discourse produces the effects that it names.”

^4^My translation of: *Der aktuelle Rechtsextremismus und Rechtspopulismus beruhen [sic!] auf einer Brutalisierung, Ethnisierung und Ästhetisierung alltäglicher Konkurrenzprinzipien.*

^5^My translation of: *Soziale Scham ist ein Machtinstrument. Leute zu beschämen ist eine der effektivsten Arten, sie still zu halten, weil sie damit die eigene untergeordnete Position internalisieren. […] Armut oder Arbeitslosigkeit werden durch die Politik der letzten Jahre immer mehr als selbstverschuldet dargestellt. Aber auch die Beschämung der sogenannten Rechtswähler als männliche, weiße Rassisten wirkt kontraproduktiv. Das hat US-Präsident Donald Trump bewiesen: Sein Angebot war: Wählt mich, und ihr müsst euch nicht mehr schämen, denn ich verkörpere alles, was man euch vorwirft. Ich verkörpere völlige Schamlosigkeit*.

^6^The only mentioning of populism is in a polemic against the accusation of shamelessness: “In the logic of The Lament, they are free to reject the specifics of Homeric *aidōs*, but must embrace its central truth: Communities need some forms of shame to survive and thrive. The populist sea must be dammed with restraints; without them, democratic peoples will take democracy *too far* in the direction of calling every hierarchy into question; the traditions of the past become entirely up for grabs.”

^7^My translation of *von außen aufoktroyier[t]*.

^8^My translation of *Ordnungsfunktion [zur] Abwehr von Angst und Verwirrung*.

^9^A different parliamentary speech by Hilse is analysed in Leser/Pates 2022, 440–442.

^10^My translation here and in the following.

^11^My translation of: *Hitler und die Nazis sind nur ein Vogelschiss in über 1000 Jahren erfolgreicher deutscher Geschichte.*

^12^My translation of: *Lieben Sie Scholten, Jelinek, Häupl, Peymann, Pasterk … oder Kunst und Kultur?*
