# Supplementary material for: Staging enmity: reading populist productions of shame with Jelinek’s On the Royal Road
Source: Open Res Eur. 2023 Jul 26;3:23. Originally published 2023 Feb 1. [Version 2] doi: 10.12688/openreseurope.15469.2 (PMC10477726; doi:10.12688/openreseurope.15469.2)
Supplement: Peer review history

# Peer review: Reviewer response for version 2

**DOI:** 10.21956/openreseurope.17669.r34018

**Published:** 2023-09-04

**Authors:** Paolo Gerbaudo

**Affiliations:** 1 Complutense University of Madrid, Madrid, Community of Madrid, Spain

This article is an interesting discussion of the way Austrian playwright Jelinek's play's On the Royal Road illuminates some of the fundamental tendencies of populism and the way populism movements revolve around the construction of a sense of we based on a strong antagonism against an "Other", identified either with immigrants, the left, elites or intellectuals. The paper manages to bridge the literature in political science and political philosophy on populism with the very different approaches and debates in the field of literary criticism and to use the work of Jelinek as a way to unearth some of the questions political scientists are trying to grapple with. The dialogue between this two areas of literature is the most interesting aspect of the paper. Central to the article is the notion of shame and shamelessness; this has been widely discussed among political theorists of populism as central to the process of identity-building of populist movements, and this very issue is also a centre-piece in Jelinek's work. In this regard the final sentence encapsulates well the message the playwright offers to scholars "The play suggests that shaming populists is everything but the royal road to limiting the detrimental effects of populist discourse—audiences’ joy in the spectacle of shame-game, on the contrary, feed into the populist production of enmity". This is an interesting take as it highlights how shame far from being a weapon for critics of populism can only end up providing further resources to populist movements and validate their opposition of us and them. More generally, the article is effective in showing how different positions of Jelinek's play problematise some of deep-seated assumptions about populism in both media and academic debate. While personally I do not totally agree with Jelinek's framing of populism, neither necessarily does the article's author. The article rather tries to use the drama as a way to read the phenomenon of populism and the debate around populism. On this basis I think that the article makes a good contribution to debates, and that the author has satisfactorily addressed the comments made by reviewers.

If any, are all the source data and materials underlying the results available?

Yes

Is the work original in terms of material and argument?

Yes

Is the work clearly and cogently presented?

Yes

Is the argument persuasive and supported by evidence?

Yes

Does the research article contribute to the cultural, historical, social understanding of the field?

Yes

Does it sufficiently engage with relevant methodologies and secondary literature on the topic?

Yes

Reviewer Expertise:

Political science and sociology; political communication; social movements and political parties; populism

I confirm that I have read this submission and believe that I have an appropriate level of expertise to confirm that it is of an acceptable scientific standard.

# Peer review: Reviewer response for version 2

**DOI:** 10.21956/openreseurope.17669.r33995

**Published:** 2023-08-01

**Authors:** Bécquer Seguín

**Affiliations:** 1 Johns Hopkins University, Baltimore, MD, USA

Thank you for the thoughtful edits. I have no further comments.

If any, are all the source data and materials underlying the results available?

Yes

Is the work original in terms of material and argument?

Yes

Is the work clearly and cogently presented?

Yes

Is the argument persuasive and supported by evidence?

Yes

Does the research article contribute to the cultural, historical, social understanding of the field?

Yes

Does it sufficiently engage with relevant methodologies and secondary literature on the topic?

Yes

Reviewer Expertise:

I am a scholar of Spanish and comparative literature. I am also a scholar of political theory, with a specific focus on populism. I have a particular expertise in the relationship between populism and literature.

I confirm that I have read this submission and believe that I have an appropriate level of expertise to confirm that it is of an acceptable scientific standard.

# Peer review: Reviewer response for version 1

**DOI:** 10.21956/openreseurope.16725.r33042

**Published:** 2023-07-10

**Authors:** Encarnacion Hidalgo Tenorio

**Affiliations:** 1 University of Granada, Granada, Spain

The author writes a beautiful text, whose main topic is not new to the reader; the focus on a theatrical play is, though. Populism and emotion have been analysed in previous literature, nevertheless, it is still very relevant, given what is going on in the world. Despite including a section on discourse analysis, the paper rather looks a content-analysis research, or a literary criticism approach to the piece, which does not have to be negatively assessed initially, but which is to the reader somewhat unexpected. Some statements should be refined; for instance, although it is true that "hatred and enmity are seminal elements of right-wing populisms", left-wing populisms also resort to these emotions; more emotion-based references on populisms could improve the final version. All in all, what is more outstanding is that the reader misses a clear set of research questions and research hypotheses, a more precise methodological section, so that this paper may be replicated in the future, as well as a final section with some concluding remarks, plus potential weaknesses.

If any, are all the source data and materials underlying the results available?

Yes

Is the work original in terms of material and argument?

Partly

Is the work clearly and cogently presented?

Yes

Is the argument persuasive and supported by evidence?

Partly

Does the research article contribute to the cultural, historical, social understanding of the field?

Yes

Does it sufficiently engage with relevant methodologies and secondary literature on the topic?

Yes

Reviewer Expertise:

Corpus-based critical discourse analysis, systemic functional linguistics, political discourse, emotion

I confirm that I have read this submission and believe that I have an appropriate level of expertise to confirm that it is of an acceptable scientific standard, however I have significant reservations, as outlined above.

# Response

**Published:** 2023-07-18

**Authors:** Juliane Prade-Weiss

**Affiliations:** Ludwig Maximilian University of Munich, Germany

Thank you very much for your insightful criticism of my article that has helped to improve it! I have addressed all issues that you have pointed out:

 - I have changed the methods-section of the abstract to make readers aware of the fact that this is a piece of literary criticism.

 - I have added a paragraph in the introduction that more clearly states the research question, my hypothesis, and - again - the literary criticism approach. I have also added research on populism to point out the lacuna that the article addresses.

 - Your comments on methodology point to a disciplinary difference, and I ask for your cordial tolerance with it: Expounding the literary criticism method further - i.e., the hermeneutical assumptions underlying close readings - would require a discussion of the philosophy of hermeneutics that far exceeds the limits of a paper. Addressing it only superficially by naming relevant authors would seem crude to peers in literary criticism, therefore, it is only done in monographs in my field, not in papers - not beyond the scope that I have pointed out in the methodology section. Moreover, there is no specific literary criticism method for engaging with populism (yet). Still, replicating the paper is not endangered by this because it hinges on reading the passages from the play that I quote, and the social sciences context I point out.

 - I have differentiated the sentence on hatred and enmity to make clear that it pertains to all populisms. The focus on right-wing populism is a demand of the other reviewer.

 - I have added research literature to support my reading of the evocation of shame.

 - I have added a concluding paragraph that points out limitations, alleys for further research by adding research literature, and a conclusion of the argument.

# Peer review: Reviewer response for version 1

**DOI:** 10.21956/openreseurope.16725.r30813

**Published:** 2023-03-15

**Authors:** Bécquer Seguín

**Affiliations:** 1 Johns Hopkins University, Baltimore, MD, USA

The present article, “Staging enmity: reading populist productions of shame with Jelinek’s *On the Royal Road*,” makes a significant contribution to scholarship on populism and literature. I recommend approving its publication with only minor changes. In this report, I will explain my reasoning for the acceptance and spell out some of the article’s contributions to the very small, yet hopefully growing body of scholarship on populism and literature. My report will first give a summary of the article, focusing on its main arguments and contributions, before going into a series of comments and, finally, providing a brief list of corrections for typos and minor errors.

 The article examines how populism discursively uses shame and shamelessness to divide citizens into camps of “us” and “them.” It then turns to literature in order to reveal the various contradictions around shame involved in both the populist discourse as well as the anti-populist discourse that seeks to challenge it. At the center of the article is a sophisticated analysis of the Austrian Nobel Prize-winning writer Elfriede Jelinek’s 2017 play *On the Royal Road*. Jelinek’s play, the article argues, occupies a privileged vantage from which to examine populist uses of shame precisely because shame is a profoundly theatrical emotion. Perhaps the most important feature of shame’s theatricality is the centrality of the audience: breaching standards, norms, and expectations can only elicit shame when doing so involves confronting an audience.

 A significant part of the article’s originality comes from its methodologically innovative treatment of populism vis-à-vis literature. The article focuses on how Jelinek’s play responds to populism, not on how it merely represents it. This may sound like a minor point, but it amounts to a strikingly novel contribution to the field. Literary studies most often focuses on questions of literary representation, in other words, on how literature portrays certain themes, historical events, etc. In fact, of the little scholarship that exists so far on literature and populism, practically all of it focuses on questions of literary representation. This is especially true in German scholarship, where scholars such as Marcus Twellmann have used contemporary accounts of populism to understand literary representations of rural life in the work of eighteenth- and nineteenth-century writers. (Cf. “Populismus und Landliteratur” pp. 133-52.) The methodological focus on representation is understandable. However, the present article importantly expands this methodological toolkit. It goes well-beyond classical accounts of Darstellung to argue instead that literature is actively engaged with contemporary political movements, it does not merely portray them. Literature, too, this article argues, wrestles with the contradictions of political movements as well as the responses to them precisely by clarifying their internal logic.

 This article, however, not only contributes to literary studies but also to scholarship on populism itself. Scholars of populism typically focus on how populism discursively frames political and other questions in stark moral terms. But this article shows just how that moralism is translated into the complex emotion of shame, which works similarly but at a different register than moral questions. In this way, the article also does a very good job of underscoring the constant contradictions that exist both on the side of the populism divide, those who support as well as those who oppose populism. Both sides operate according to a similar logic that produces stark moral divides between in-group and out-group members. The play, the article argues, is also an attempt to clarify just how much of the populist logic much anti-populist discourse reproduces.

 I will now turn to several comments, some critical, about the article. These should not be read as disqualifying but rather as feedback meant to strengthen the argument and point out potential future criticisms.

 It strikes me that more could be done to justify the focus on shame. A scholar of literature or populism might think, Why shame now? One reason could be that shame is a standard response from liberalism to political crises. Think of the human rights movement. For many human rights organizations, ‘naming and shaming’ (e.g. public opprobrium) is the preferred mode of activism. Thus, shame is important for questions that go well-beyond populism. Shame is a general feature of left-of-center politics that deserves more scrutiny. Understanding how shame works is key to comprehending liberal politics more generally, including what motivates its supporters as well as its profound limitations. Making this, or a similar justification explicitly would go a long way toward highlighting the importance of this scholarly focus for readers.

 When speaking about populism, the article clearly appears to have right-wing populism in mind—as opposed to left-wing populism, e.g., Die Linke. Perhaps this focus on the right, as opposed to the left, could be made more explicit, if only to hedge against potential critics who would not agree, for example, that “conspiracy myths are a structural necessity” (5) for left-wing populism. Conversely, if one of the claims of the article is to show that there does not, in fact, exist a meaningful difference between left- and right-wing populism, then the article could explain why via Müller or Mudde & Kaltwasser, for example.

 On page 10, the article brings up the question of egalitarianism. I am very skeptical that populists, whether right- or left-wing, see themselves “egalitarian.” Democratic, certainly. But not egalitarian. This is because egalitarian principles of equality are often associated with the parts of liberal rights discourse that protect minority rights. Populists, by contrast, often view themselves as defending majority rights against entrenched minority interests or other mechanisms in liberal democracies that prevent the democratic majority from expressing its political will.

 Throughout the article, the phrase “populist production” regularly appears. This phrase is admittedly confusing. Reading the article, I often found myself unsure of whether what was being referred to was Jelenik’s play, or the theatricality of populist movements, or both. Although I understand that the article analyzes how a play thematizes populism, I think avoiding such a term would help clarify the argument by making explicit when the literary work is being analyzed and when populism more generally is being analyzed.

 The final page of the article struck me as so clear and powerful that I wonder whether there is any way that it might be moved up toward the beginning of the article so as to clearly convey the argument of the paper. I fear that placing this section at the end, as it is now, buries one of the best explications of the article’s argument. Placing it nearer to the beginning would also allow the article to weave together the theoretical discussion and the literary analysis more consistently, since the focus of the attention at the beginning of the article is heavily on populism.

 Finally, I wish to point out several typos and minor errors I came across while reading the manuscript. This is not an exhaustive list, so I may have missed other typos. In any case, my final suggestion is to revise the article at least once, if not several more times, so as to make sure it is free of any such similar issues. Here are my edits, with the correction in brackets:

 p. 5: “…allows [us] to follow…”

 p. 5: “…impact [on] democracies…”

 p. 8: “…by [a near-]majority of…”

 p. 9: “…it [tugs] at…”

 p. 10: “…under [Locke’s] notion…”

 p. 13: “…problem [with] the populist…”

 P. 14: “…shame-game [are] very often…”

 p. 15: “…for the [critic]…”

 Again, I wish to unequivocally state my support for the publication of this article with the minor revisions I have suggested throughout this report. The article is a welcome and significant contribution to an emerging body of scholarship that will have a lasting impact not only on how we understand the relationship between populism and literature, but also, and more generally, on the politics of literature more broadly.

If any, are all the source data and materials underlying the results available?

Yes

Is the work original in terms of material and argument?

Yes

Is the work clearly and cogently presented?

Yes

Is the argument persuasive and supported by evidence?

Yes

Does the research article contribute to the cultural, historical, social understanding of the field?

Yes

Does it sufficiently engage with relevant methodologies and secondary literature on the topic?

Yes

Reviewer Expertise:

I am a scholar of Spanish and comparative literature. I am also a scholar of political theory, with a specific focus on populism. I have a particular expertise in the relationship between populism and literature.

I confirm that I have read this submission and believe that I have an appropriate level of expertise to confirm that it is of an acceptable scientific standard.

# Response

**Published:** 2023-07-18

**Authors:** Juliane Prade-Weiss

**Affiliations:** Ludwig Maximilian University of Munich, Germany

Thank you so much for your comprehensive comments on my paper, for the support, and the criticism. The latter I have addressed as follows:

 - I have flagged more clearly that I focus on right-wing populism.

 - I have made clearer that shame is one of the dynamics at play, yet not the only relevant emotion.

 - I have altered the evocation of egalitarianism, which I took from the quote, to democracy; my aim is not to argue for more. And I have corrected the typos. Thank you very much for pointing them out!
